# Human viral nucleic acids concentrations in wastewater solids from Central and Coastal California USA

**DOI:** 10.1038/s41597-023-02297-7

**Published:** 2023-06-22

**Authors:** Alexandria B. Boehm, Marlene K. Wolfe, Krista R. Wigginton, Amanda Bidwell, Bradley J. White, Bridgette Hughes, Dorothea Duong, Vikram Chan-Herur, Heather N. Bischel, Colleen C. Naughton

**Affiliations:** 1 Department of Civil & Environmental Engineering, School of Engineering and Doerr School of Sustainability, Stanford University, Stanford, CA USA; 2 Gangarosa Department of Environmental Health, Rollins School of Public Health, Emory University, Atlanta, GA USA; 3 Department of Civil and Environmental Engineering, University of Michigan, Ann Arbor, 48109 Michigan USA; 4 Verily Life Sciences, South San Francisco, CA USA; 5 Department of Civil and Environmental Engineering, University of California Davis, Davis, CA 95616 USA; 6 Department of Civil and Environmental Engineering, University of California Merced, Merced, CA 95343 USA

**Keywords:** Infectious diseases, Environmental sciences

## Abstract

We measured concentrations of SARS-CoV-2, influenza A and B virus, respiratory syncytial virus (RSV), mpox virus, human metapneumovirus, norovirus GII, and pepper mild mottle virus nucleic acids in wastewater solids at twelve wastewater treatment plants in Central California, USA. Measurements were made daily for up to two years, depending on the wastewater treatment plant. Measurements were made using digital droplet (reverse-transcription–) polymerase chain reaction (RT-PCR) following best practices for making environmental molecular biology measurements. These data can be used to better understand disease occurrence in communities contributing to the wastewater.

## Background & Summary

Wastewater-based epidemiology uses concentrations of infectious disease targets in wastewater to understand disease occurrence in communities. Before the COVID-19 pandemic, it had been used to understand poliovirus circulation^1^, as well as infections associated with enteric pathogens like *Salmonella*^2^.

Wastewater represents a composite biological sample that contains contributions from every individual using drains and toilets in the sewershed. Human excretions including feces, saliva, urine, blood, and mucus all enter the wastewater system. When individuals are infected with a pathogen, they excrete the pathogen (infectious or non-infectious) via excretions that contribute to wastewater. Although the amount of pathogen shed by an individual may depend on disease severity or progression of illness, and whether the individual has an asymptomatic or symptomatic infection, all individuals, regardless of disease status, contribute to wastewater. This means that wastewater monitoring can be a low-bias approach to understanding disease occurrence in communities. Information gleaned from wastewater monitoring can complement clinical specimen testing data which may be biased owing, in part, to testing availability and test-seeking behaviors of individuals^3–5^.

**Fig. 1:**
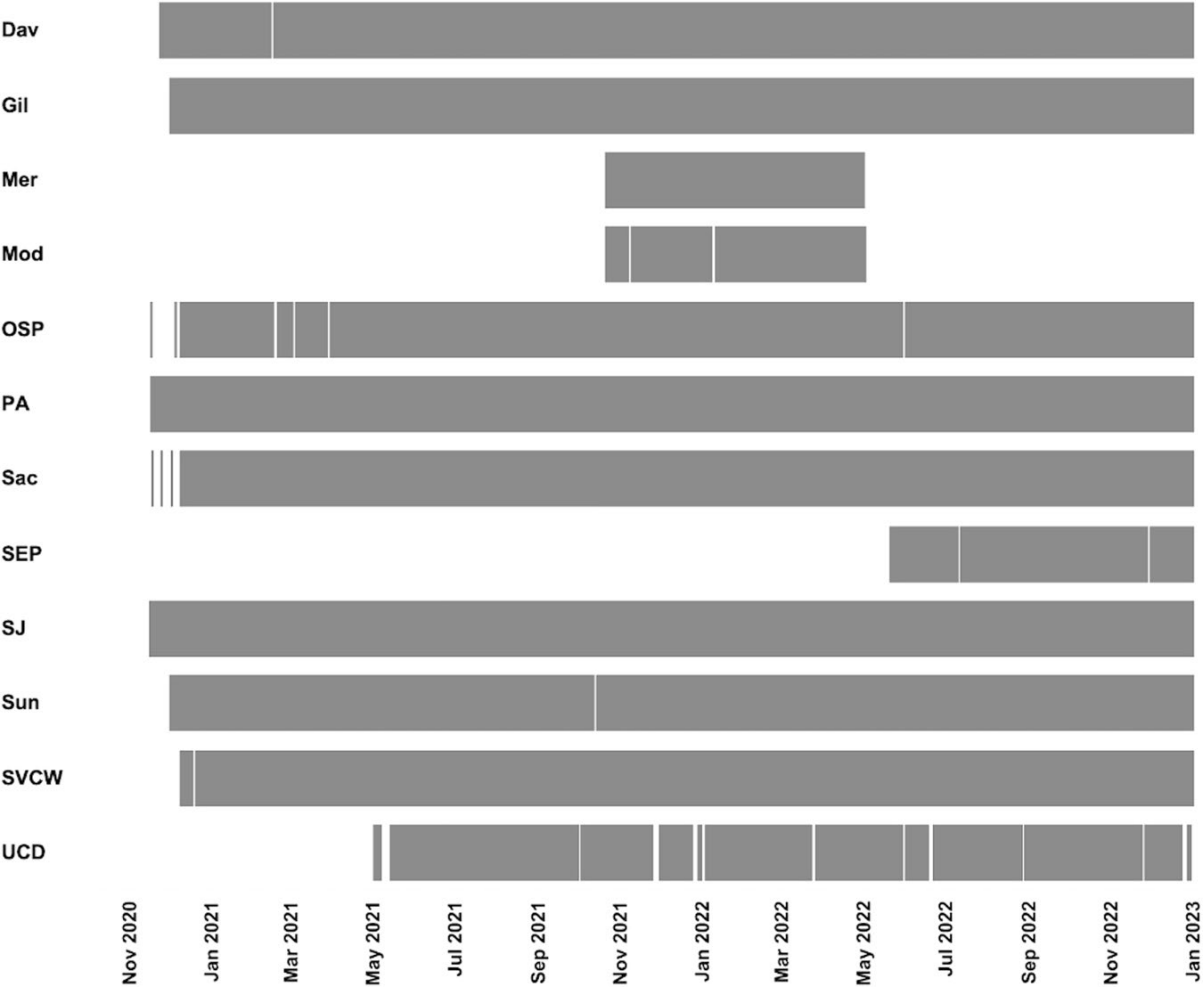
The time period during which each of the 12 plants were enrolled in the study. Each plant is a row on the y-axis, and date is on the x-axis. When a sample was collected, gray is shown; white indicates no sample collected. The abbreviation for each plant is provided in Table 1.

Early research at the start of the COVID-19 pandemic suggested that SARS-CoV-2 RNA partitioned to wastewater solids where it could be found in concentrations 1,000–10,000 times higher than liquid wastewater^6^, and that concentrations of SARS-CoV-2 in wastewater solids correlated well with incident, reported COVID-19 cases in communities contributing to the wastewater^7,8^. Given these findings, we implemented the Sewer Coronavirus Alert Network (SCAN) project to provide real time data on concentrations of SARS-CoV-2 RNA in wastewater solids samples in late 2020. The project ended in December 2022 when it was replaced by a larger national project WastewaterSCAN. Up to twelve wastewater treatment plants (WWTPs) were enrolled in SCAN and provided samples of wastewater solids daily. We processed the samples and made data available within 24 hours of sample receipt at the laboratory. Initially, we measured three genes found in the SARS-CoV-2 genome (N, S and ORF1A) as well as a gene in the pepper mild mottle virus (PMMoV). PMMoV is highly abundant in human stool and domestic wastewater globally^9^ and is used here as an internal recovery and fecal strength control^10,11^. We later added additional SARS-CoV-2 genomic targets that detected mutations that were characteristic of circulating variants of concern. Additional work by our group showed the that influenza A and B viruses, respiratory syncytial virus, mpox virus, and human metapneumovirus nucleic acids in wastewater solids was well associated with data on incident cases in the contributing communities^5,12–14^, so we added measurements for these into the SCAN program as well. We additionally added measurement for norovirus GII given evidence its presence in wastewater also relates to community disease occurrence^15^. A diagram illustrating the enrollment of WWTPs and duration of measurements in the program is provided in Fig. 1.

We are making these data available for others to use to better understand disease circulation in communities. Researchers have suggested that wastewater data can be used to predict the reproductive number of a disease^16^, and predict incident cases^17^, and hospitalizations^18^. We anticipate future work with our and others’ data will provide new insights into the utility of wastewater surveillance for understanding disease epidemiology.

## Methods

### Sampling locations

**Fig. 2:**
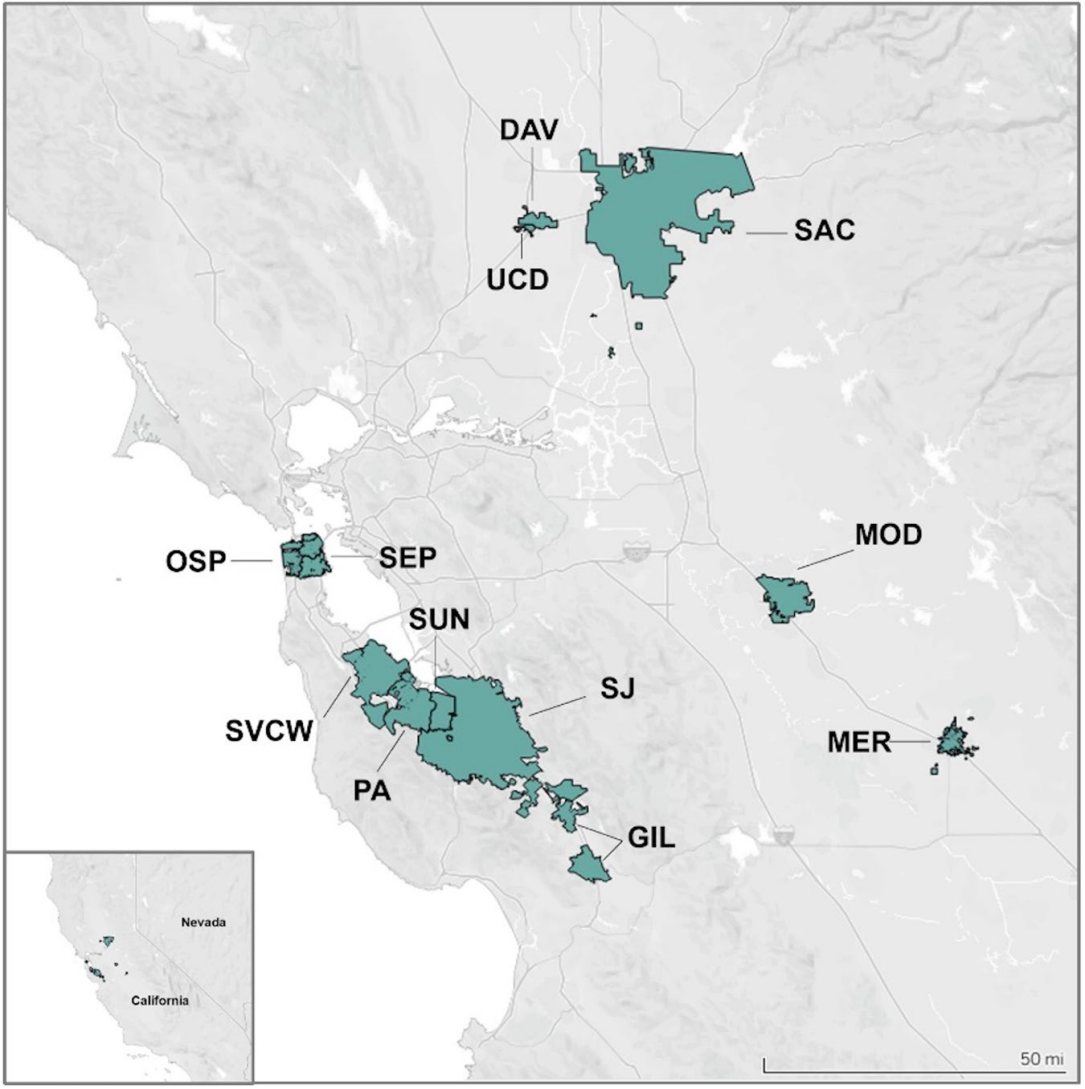
Maps of sewersheds of wastewater treatment plants enrolled in this study.

At any one time, between eight and twelve WWTPs were enrolled in the SCAN project (Table 1, Figs. 1, 2). WWTPs served between 30,000 and 1,500,000 people and were located in Santa Clara, San Mateo, San Francisco, Sacramento, Yolo, Merced, and Stanislaus Counties, California, USA. WWTPs participated in SCAN for between 185 and 777 days (median = 752 days) between 15 Nov 2020 and 31 Dec 2022.

**Table 1:** Details of sampling locations and time period of sampling.

WWTP name	WWTP acronym	County	Population served	First sample date	Last sample date (m/d/y)	Number of samples collected
Davis Wastewater Treatment Plant	Dav	Yolo	68,000	11/23/20	12/31/22	766
South County Regional Wastewater Authority	Gil	Santa Clara	110,338	11/30/20	12/31/22	760
City of Merced Wastewater Treatment Plant	Mer	Merced	91,000	10/20/21	4/30/22	191
Modesto Wastewater Treatment	Mod	Stanislaus	230,000	10/20/21	5/01/22	185
Oceanside Water Pollution Control Plant	OSP	San Francisco	250,000	12/4/20	12/31/22	722
Palo Alto Regional Water Quality Control Plant	PA	Santa Clara	236,000	11/16/20	12/31/22	770
Sacramento Regional Wastewater Treatment Plant	Sac	Sacramento	1,480,000	11/17/20	12/31/22	755
San José-Santa Clara Regional Wastewater Facility	SJ	Santa Clara	1,500,000	11/15/20	12/31/22	777
Silicon Valley Clean Water	SVCW	San Mateo	199,000	12/8/20	12/31/22	749
Sunnyvale Solid Waste Management	Sun	Santa Clara	153,000	11/30/20	12/31/22	756
Southeast Water Pollution Control Plant	SEP	San Francisco	750,000	5/20/22	12/31/22	221
University of California - Davis Wastewater Treatment Plant	UCD	Yolo	30,000	11/21/20	12/29/22	558

WWTP stands for wastewater treatment plant.

### Sample collection

Settled solids from wastewater were collected from WWTPs by dedicated staff each day. Samples were collected using sterile technique in clean, labeled bottles provided by our team. WWTPs were not provided compensation for participation. Samples were “grab” samples from the primary clarifier at the plants with the following exceptions: 1) SJ provided a manually collected 24-hour composite sample from the primary clarifier by collecting subsample every 6 hours; 2) UCD and Gil collect a 24-hour composite sample from their influent and then let it settle to generate a sample of settled solids following Standard Method 160.5^19^ for Gilroy and Montesinos-López *et al*.^20^ for UCD. A total of 7,210 samples were collected for this study.

WWTPs finished their sample collection in the morning of each day and labeled the sample with the date when they cap the sample with the following exceptions. 1) PA collected their grab sample at 14:00 the day before the sample was picked up because that is the time of day when their primarily clarifier settled solids waste stream is thickest and when they had staff available to collect the sample. They then held the sample at 4 °C until it was picked up the next day; the sample was labeled with the date it was collected. 2) SJ labeled their sample with the previous day’s date because that was the date for which the majority of the composite sample was collected. We note that the “grab” samples mentioned above are in fact solids from the wastewater that are collected over 1–6 hours in the clarifiers. They are therefore essentially a composite of wastewater solids from the community over several hours.

Samples were immediately stored at 4 °C and transported in a cooler with ice packs to the commercial partner laboratory by a courier service where processing began within 6 h of collection.

### Pre-analytical processing

The methods for pre-analytical processing are described step-by-step in a protocol on protocols.io^21^ and some of these methods are presented in Wolfe *et al*.^22^ The solids were dewatered by centrifugation at 24,000 × g for 30 min at 4 °C. The supernatant was aspirated and discarded. A 0.5- to 1-g aliquot of the dewatered solids was dried at 110 °C for 19 to 24 h to determine its dry weight. Bovine coronavirus (BCoV) was used as a positive recovery control. Each day attenuated bovine coronavirus vaccine (PBS Animal Health, Calf-Guard Cattle Vaccine) was spiked into DNA/RNA shield solution (Zymo Research) at a concentration of 1.5 μl/ml. Dewatered solids were resuspended in the BCoV-spiked DNA/RNA shield to a concentration of 75 mg/ml. This concentration of solids was chosen as previous work titrated solutions with various concentrations of solids to identify a concentration that minimized inhibition while maintaining sensitivity of SARS-CoV-2 assays^16^. Between 5 and 10 stainless steel grinding balls (5/32-in., OPS Diagnostics) were added to each sample, which was subsequently homogenized by shaking with a Geno/Grinder 2010 (Spex SamplePrep). Samples were then briefly centrifuged to remove air bubbles introduced during the homogenization process. Before 29 May 2021, the samples were then vortexed to remix the sample and aliquots were used directly for nucleic-acid extraction. Starting 29 May 2021, we omitted the final vortexing step.

### Nucleic-acid extraction

The methods for nucleic acid extraction are described step-by-step in a protocol on protocols.io^23^ and in Wolfe *et al*.^22^ Nucleic-acids were extracted from 10 replicate aliquots per sample. For each replicate, nucleic-acids were extracted from 300 μl of homogenized sample using the Chemagic Viral DNA/RNA 300 kit H96 for the Perkin Elmer Chemagic 360 followed by PCR inhibitor removal with the Zymo OneStep-96 PCR Inhibitor Removal kit. Extraction-negative controls (water) and extraction-positive controls were extracted using the same protocol as the homogenized samples. The positive controls consisted of different targets (Table 2) added into the BCoV-spiked DNA/RNA shield solution described above.

**Table 2:** Targets measured in the study.

Target	Approximate Start date	Approximate End date	Positive control material	Assay reference
SARS-CoV-2 N gene	11/15/20	12/31/22	SARS-CoV-2 gRNA (ATCC VR-1986D)	Wolfe *et al*.^22^
SARS-CoV-2 S gene	11/15/20	12/31/22	SARS-CoV-2 gRNA (ATCC VR-1986D)	Wolfe *et al*.^22^ and this paper
SARS-CoV-2 ORF1a gene	11/15/20	9/3/21	SARS-CoV-2 gRNA (ATCC VR-1986D)	Wolfe *et al*.^22^
RSV	1/9/22	12/31/22	Intact RSV B virus (Zepto NATFVP-NNS)	Hughes *et al*.^14^
Influenza A	1/9/22	12/31/22	Twist Synthetic Influenza H3N2 RNA Control (Twist 103002)	CDC^33^
				Wolfe *et al*.^13^
Influenza B	6/6/22	12/31/22	Twist Synthetic Influenza B RNA Control (Twist 103003)	CDC^33^
				Boehm *et al*.^5^
Norovirus GII	11/8/22	12/31/22	Quantitative Synthetic RNA from Norovirus G2 (II) (ATCC VR-3235SD)	Loisy *et al*.^34^
Human metapneumovirus	6/9/22	12/31/22	Gene block	Boehm *et al*.^5^
MPOX	6/18/22	12/31/22	Gene block	Wolfe *et al*.^12^
				Li *et al*.^35^
SARS-CoV-2 HV69-70	6/9/22	12/31/22	SARS-CoV-2 variant B.1.1.7, ATCC VR-3326HK	Yu *et al*.^31^
				Wolfe *et al*.^30^
SARS-CoV-2 del156/157	9/4/21	2/10/22	Twist Synthetic Delta gRNA control 23	Yu *et al*.^31^
				Wolfe *et al*.^30^
SARS-CoV-2 del143/145	12/6/21	2/10/22	Twist Synthetic Omicron gRNA control 48	Boehm *et al*.^32^
SARS-CoV-2 BA_2_LPPA24S	2/11/22	9/13/22	Twist synthetic Omicron BA.2 gRNA control 50	Boehm *et al*.^32^
SARS-CoV-2 BA_2_75_S_147E_S_152R	8/23/22	11/15/22	Gene block	This paper
SARS-CoV-2 BA_4_ORF1a_Del_141143	5/19/22	11/15/22	Gene block	This paper

The period of time during which we applied the assay and the positive control material used, as well as the reference for the primers and probes are provided. Approximate start and end dates represent the data of the first and last sample tested using the assay, dates are approximate because some plants may have had a sample before or after the provided date tested, actual first dates for each of the plants is provided in the data file. The vendor Twist Biosciences (“Twist”) is located in South San Francisco, CA. ATCC is American Type Culture Collection.

### Digital-droplet RT-PCR

Nucleic-acid extracts were used as the template in digital droplet RT-PCR assays. BCoV and PMMoV were quantified using a duplex assay in each sample. Over the course of the study, we added and removed different assays from the program (Table 2) as different variants of SARS-CoV-2 emerged and science became available to support the addition of various virtual nucleic-acid targets. The primers and probes used are provided in Table S1. We changed the SARS-CoV-2 S gene assay on 5 September 2021 and then again on 22 December 2021 to respond to mutations in the binding regions of our S gene assay primers and probes. We changed the MPOX virus assay on 1 December 2022 in response to the expressed preference of the United States Center for Disease Control. Undiluted nucleic-acid extract was used as template in all assays for infectious disease targets, and a 1:100 dilution of the extract was used for the BCoV/PMMoV assay template. The one exception was for Mod where for samples collected on and after 30 December 2021, we used a 1:10 dilution of the nucleic-acid extract as template to alleviate suspected RT-PCR inhibition as this plant receives some cannery wastes.

**Fig. 3:**
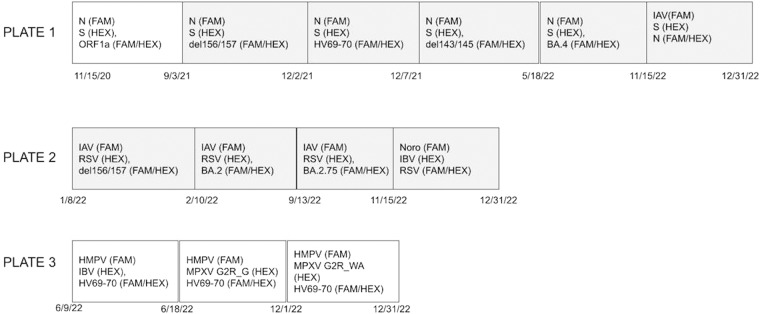
Arrangement of multiplexed PCR plates for human viral targets run in the study. Each box represents a schematic of how assays were multiplexed during different periods of the project. The date at the bottom left side of each box is the start date and the date near the right edge of the box is the approximate end date (based on the date the assay was stopped in the lab, the date associated with the last sample run could be different depending on the date it was processed in the lab). All probes contained fluorescent molecules and quenchers (5′ FAM and/or HEX/ZEN/3′ IBFQ), as indicated. FAM, 6-fluorescein amidite; HEX, hexachloro-fluorescein; ZEN, a proprietary internal quencher from IDT; IBFQ, Iowa Black FQ. If box is white, annealing temperature is 59 °C and if gray, it is 61 °C. N, S, and ORF1a are assays targeting those genes in SARS-CoV-2. del156/157 is the assay targeting the 156/157 deletion in the S gene characteristic of the Delta variant of SARS-CoV-2. HV69-70 is the assay targeting the deletion 69/70 in the S gene characteristic of Alpha, and various Omicron variants. del143/145 is the assay targeting the 143/145 deletion in the S gene characteristics of BA.1 Omicron. BA.4 is the assay targeting the deletion 141/143 in the ORF1a gene characteristic of BA.4. BA.2.75 is the assay targeting the adjunct SNPS (S:147E and) in the S gene characteristic of BA.2.75. IAV is the assay targeting a gene in the influenza A genome. RSV is the assay targeting a gene in the RSV genome. BA.2 is the assay targeting the set of deletions LPPA24S characteristic of BA.2. IBV is the assay targeting influenza B. MPXV is the assay targeting genes in MPOX virus. HMPV is the assay targeting genes in human metapneumovirus.

Digital droplet RT-PCR was performed on 20 μl samples from a 22 μl reaction volume, prepared using 5.5 μl template, mixed with 5.5 μl of One-Step RT-ddPCR Advanced kit for Probes (catalog no. 1863021; Bio-Rad), 2.2 μl reverse transcriptase, 1.1 μl dithiothreitol (DTT), and primers and probes at a final concentration of 900 nM and 250 nM, respectively. Droplets were generated using the AutoDG Automated Droplet Generator (Bio-Rad). PCR was performed using Mastercycler Pro with the following protocol: reverse transcription at 50 °C for 60 min, enzyme activation at 95 °C for 5 min, 40 cycles with 1 cycle consisting of denaturation at 95 °C for 30 s and annealing and extension at either 59 °C or 61 °C (for human viral targets, Fig. 3) or 56 °C (for PMMoV/BCoV duplex assay) for 30 s, enzyme deactivation at 98 °C for 10 min, and then an indefinite hold at 4 °C. The ramp rate for temperature changes was set at 2 °C/s, and the final hold at 4 °C was performed for a minimum of 30 min to allow the droplets to stabilize. Droplets were analyzed using the QX200 Droplet Reader (Bio-Rad). All liquid transfers were performed using the Agilent Bravo (Agilent Technologies). These methods are also provided by Wolfe *et al*.^22^.

Assays for human viral targets were run in triplex using the triplex probe mixing approach^24^. The manner in which the assays were triplexed changed over time as we added and removed assays to detect different viruses of public health importance. We began with one set of three assays run in triplex on a single 96-well plate at the beginning of the study, and by the end of the study we were running three sets of three assays (9 assays total) using three 96-well plates. Figure 3 shows how assays were triplexed over the duration of the study.

Each sample was run in 10 replicate wells, extraction-negative controls were run in 3 to 7 wells, and extraction-positive controls in 1 well. In addition, PCR-positive controls were run in 1 well, and no-template controls (NTC) (negative PCR controls) were run in 3 to 7 wells. The positive controls that were used are provided in Table 2. Results from replicate wells were merged for analysis. Thresholding was done using QuantaSoft Analysis Pro software (Bio-Rad, version 1.0.596). In order for a sample to be recorded as positive, it had to have at least three positive droplets. Three positive droplets corresponds to a concentration between ∼500 and 1000 copies (cp)/g; the range in values is a result of the range in the equivalent mass of dry solids added to the wells.

Concentrations of nucleic-acid targets were converted to concentrations per dry weight of solids in units of cp/gram (dry weight) using dimensional analysis. The total error is reported as 68% confidence intervals and includes the errors associated with the Poisson distribution and the variability among the 10 replicates. Total error for the merged replicates is reported as output by the QuantaSoft Analysis Pro software. The recovery of BCoV was determined by normalizing the concentration of BCoV by the expected concentration given the value measured in the BCoV-spiked DNA/RNA shield. BCoV recovery was used solely as a process control and not used in the calculation of concentrations^25^.

### RT-PCR assay design

The design of all assays included in the study have been described previously in peer-reviewed publications with the exception of the assays for the two revised S gene assays (rev and rev2 in Table S1), and the assays for detecting mutations characteristic of BA.2.75 and BA.4, labeled SARS-CoV-2 BA_2_75_S_147E_S_152R and SARS-CoV-2 BA_4_ORF1a_Del_141143, respectively, in Table 3. To design these assays, sequences of circulating variants (in the case of the S gene assay) or of the variant of interest (BA.4 or BA.2.75) were downloaded from the National Center for Biotechnology Information (NCBI) in the month prior to assay deployment and aligned to identify conserved regions. Assays were developed *in silico* using Primer3Plus (https://www.primer3plus.com/). Parameters used in assay development (e.g., sequence length and GC content) are provided elsewhere^5^. Primers and probes were screened for specificity *in silico*, and *in vitro* against virus panels, intact respiratory viruses, synthetic viral genomic RNA, or cDNA sequences (Table 3).

**Table 3:** List of new assays presented in this study, and the name of the gene target RT-PCR probe-based assays targeted.

Assay name	Gene target region	Non-target testing (specificity)	Target testing (sensitivity)
SARS-CoV-2 S gene (rev)	S gene	SARS-CoV-2 gRNA (ATCC VR-1986D), Twist synthetic gRNA for Alpha, Beta	Gene Block, Twist Synthetic gRNA for Delta
SARS-CoV-2 S gene (rev 2)	S gene	SARS-CoV-2 gRNA (ATCC VR-1986D), Twist Synthetic gRNA for Delta, Mu, Lambda, Beta	Twist Synthetic gRNA for Omicron BA.1
SARS-CoV-2 BA_4_ORF1a_Del_141143	ORF1a	SARS-CoV-2 gRNA (ATCC VR-1986D), Twist Synthetic gRNA for Omicron BA.1, Omicron BA.2, and Delta	Gene block
SARS-CoV-2 BA_2_75_S_147E_S_152R	S gene	SARS-CoV-2 gRNA (ATCC VR-1986D, Twist Synthetic gRNA for Omicron BA.1, Omicron BA.2	Gene block

The list of non-target and target controls used to test assay specificity and sensitivity are provided. Gene blocks are dsDNA molecules with the target sequence purchased from Integrated DNA Technologies (IDT, Coralville, Iowa).

## Data Records

All measurements made in this study are available at a permanent URL at the Stanford Digital Depository: 10.25740/cx529np1130^26^. The data are available in a CSV file called “SCAN_AllPlants_SDR_7April23.csv” that includes the fields in the list below as columns.

Plant Abbr: This is the plant abbreviation (see Table 1)

Month: This is a month associated with the sample

Day: This is the day associated with the sample

Year: This is the year associated with the sample.

N_Gene_gc_g_dry_weight: Concentration of the N gene in gene copies per mass of wastewater solids as measured by dry weight.

ORF1a_gc_g_dry_weight: Concentration of the ORF1a gene in gene copies per mass of wastewater solids as measured by dry weight.

S_Gene_gc_g_dry_weight: Concentration of the S gene in gene copies per mass of wastewater solids as measured by dry weight.

Delta_156157_gc_g_dry_weight: Concentration of the Delta mutation (deletion 156/157 in the S gene) in gene copies per mass of wastewater solids as measured by dry weight.

Del_143145_gc_g_dry_weight: Concentration of the Omicron BA.1 mutation (S:del143/145 in the S gene) in gene copies per mass of wastewater solids as measured by dry weight.

HV_6970_Del_gc_g_dry_weight: Concentration of the HV69–70 mutation (S:del69/70 in the S gene) in gene copies per mass of wastewater solids as measured by dry weight.

BA_4_ORF1a_Del_141143_gc_g_dry_weight: Concentration of the ORF1A:del141/143 mutation (found in BA.4) in gene copies per mass of wastewater solids as measured by dry weight.

Influenza_B_gc_g_dry_weight: Concentration of the Influenza B gene in gene copies per mass of wastewater solids as measured by dry weight.

Influenza_A_gc_g_dry_weight: Concentration of the Influenza A gene in gene copies per mass of wastewater solids as measured by dry weight.

RSV_gc_g_dry_weight: Concentration of the RSV gene in gene copies per mass of wastewater solids as measured by dry weight. The assay detects both RSV A and RSV B.

BA_2_LPPA24S_gc_g_dry_weight: Concentration of the S:LPPA24S mutation (a 9 bp deletion in the S gene) in gene copies per mass of wastewater solids as measured by dry weight.

HMPV_4_gc_g_dry_weight: Concentration of the human metapneumovirus gene in gene copies per mass of wastewater solids as measured by dry weight.

Noro_G2_gc_g_dry_weight: Concentration of a gene in norovirus GII in gene copies per mass of wastewater solids as measured by dry weight.

MPXV_G2R_G_gc_g_dry_weight: Concentration of the monkeypox gene measured with the G2R_G assay in gene copies per mass of wastewater solids as measured by dry weight.

MPXV_G2R_WA_gc_g_dry_weight: Concentration of the monkeypox gene measured using the G2R_WA assay in gene copies per mass of wastewater solids as measured by dry weight.

BA_2_75_S_147E_S_152R_gc_g_dry_weight: Concentration of the characteristic adjacent mutations in BA.2.75 (S:K147E and S:W152R) in gene copies per mass of wastewater solids as measured by dry weight.

PMMoV_gc_g_dry_weight: Concentration of PMMoV in gene copies per mass of wastewater solids as measured by dry weight.

BCoV_Recovery: fractional recovery between 0 and 1 of Bovine coronavirus. Sometimes the value will be greater than 1 owing to uncertainty in the denominator of the ratio.

### Additional variables

All variables ending in **_UCL** represent the upper 68% confidence limit of the variable.

All variables ending in **_LCL** represent the lower 68% confidence limit of the variable.

If a cell is blank, it means the assay was not run for that day.

If a “0” appears, it means the assay was a non-detect. The detection limit varies by sample depending on the amount of solids by dry weight included, but is between 500–1000 cp/g dry weight.

## Technical Validation

The analyses followed the Minimum Information for Publication of Quantitative Digital PCR Experiments (dMIQE2020 guidelines) published by Huggett *et al*.^27^ and the Environmental Microbiology Minimum Information (EMMI) guidelines published by Borchardt *et al*.^28^. Checklists for both can be found in the Supplementary Information (Supplementary Information Table S2 and Figure S1).

Strict QA/QC procedures (including negative and positive extraction and PCR controls for all targets, and recovery controls) coupled to replicate analyses (n = 10) for each sample ensured high-quality data. As mentioned in the methods, negative and positive extraction and RT-PCR controls were run on each plate. In order for a plate to pass QA/QC, there had to be 2 or fewer droplets across all negative controls, and positive controls had to have 3 or more droplets. All data included in this study passed these QA/QC metrics. If a plate did not meet these QA/QC criteria, then all the samples were re-run.

**Fig. 4:**
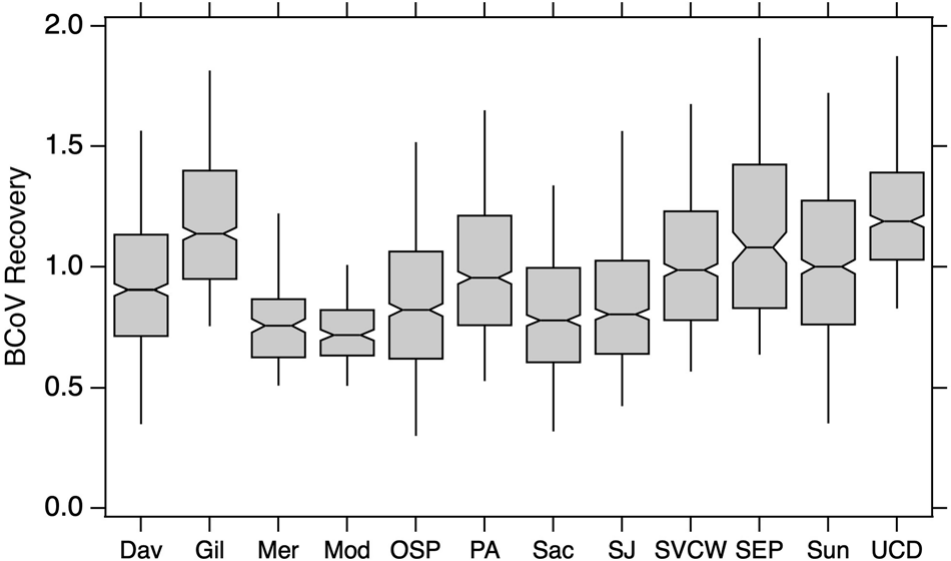
Bovine coronavirus recovery at each plant. The lower and upper edges of the boxes are the 25th and 75th percentiles, respectively. The middle line in each box is the median. The lower and upper “whiskers” end at the 9th and 91st percentiles. The number of recoveries used for each plant are provided in Table 1. Abbreviations for each plant are shown on the x-axis. Multiple the factional values by 100 to obtain recovery as a percent.

Median bovine coronavirus (BCoV) recoveries were, on average 93% (25th percentile = 72%, 75th percentile = 120%) across all 7210 samples, and all were above 1%. This indicates lack of gross inhibition and good and consistent recovery of viral nucleic during extraction. Figure 4 shows a box and whisker plot of BCoV recoveries across WWTPs. At times, a recovery higher than 100% was recorded, likely a result of underestimation of the amount of BCoV spiked into the samples. We did not attempt to correct any of our measurements by BCoV recovery owing to the complexities associated with estimating endogenous viral recovery in environmental matrices^25^.

Details associated with the Minimal Information MIQE Experiments (dMIQE) for digital droplet PCR reporting are described here. The average (standard deviation) number of droplets in ten merged wells determined from a random subset of results of 200 samples was 180000 (1800). Average number of copies per partition (λ) (standard deviation) for SARS-CoV-2 N gene in the same subset was 2.0 × 10^−3^ (1.1 × 10^−4^), for PMMoV 0.18 (8.8 × 10^−4^), and for BCoV 4.3 × 10^−3^ (1.5 × 10^−4^). λ for other human viruses was similar to that for SARS-CoV-2 N gene.

Sample standard deviations for the measured targets were 11% (median, IQR: 5%–25%) of the measurement. As the samples were extracted 10 times and each extract was analyzed in 1 of 10 replicate wells which were merged, the replicate variability incorporates variation from both nucleic-acid extraction and reverse transcription-digital droplet PCR (RT-ddPCR) with a heterogeneous solid sample. Assays were conducted in only one lab, so reproducibility was not assessed.

The theoretical lowest measurable concentration was 3 positive droplets which translates into between 500 and 1000 copies/g dry weight depending on the percent dry weight of the solids used in the extraction which depends on the properties of the solid and how effectively it can be dewatered.

Example fluorescence plots from the digital PCR machine can be found in Wolfe *et al*.^22^ and Topol *et al*.^29^.

As mentioned in the methods, we found that using a solids concentration of 75 mg of solids per ml of DNA/RNA shield during the pre-analytical process was sufficient to alleviate inhibition while allowing good sensitivity of the assay (adding too little solids would result in a relatively high lower detection limit which is not ideal). During the prospective wastewater monitoring described in this Data Descriptor, we tested inhibition quarterly in samples. To do so, we titrated the solids concentration using 7.5 mg/ml, 15 mg/ml, 37.5 mg/ml, 75 mg/ml, and 150 mg/ml and compared the resultant measured concentrations (calculated considering the different solid concentrations used). If the RT-PCR were inhibited at 75 mg/ml, one would expect concentrations measured using lower concentrations of solids would be higher that those measured using 75 mg/ml.

**Fig. 5:**
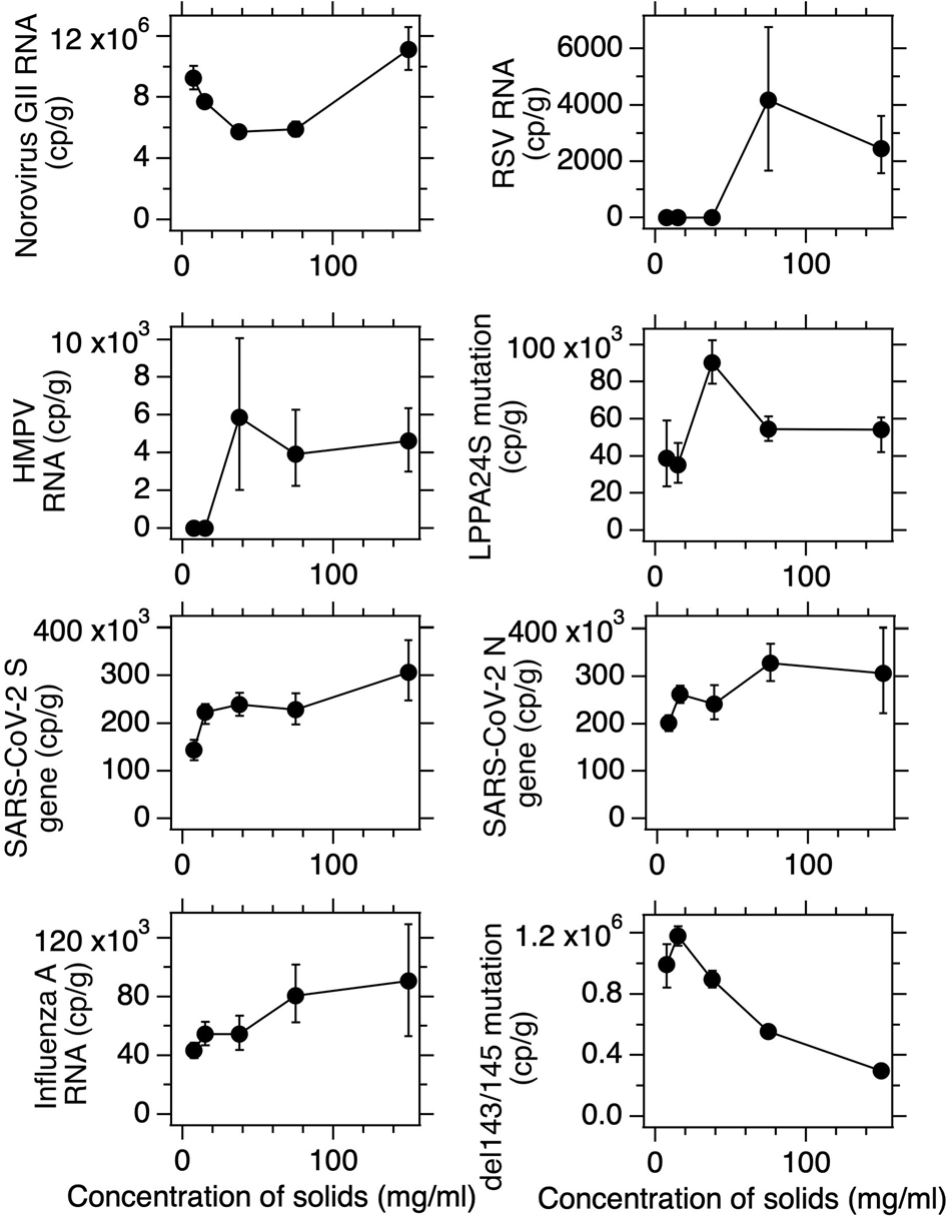
Example inhibition titrations for eight assays used for the study. The concentration of solids from wastewater solids samples placed in DNA/RNA Shield in the preanalytical step prior to homogenization and RNA extraction is shown on the x-axis. On the y-axis is the concentration of the target measured in the solids in units of copies per gram (cp/g) dry weight, corrected for the concentration of solids placed in the DNA/RNA shield. If a symbol is on the value of “0”, it means the result was “not detected”. This occurred in some cases when the mass of solids added to the DNA/RNA shield was small, thereby reducing the sensitivity of the method. Errors are shown as 68% confidence intervals across 10 replicates as described in the methods section. If error bars are not visible it is because they are smaller than the symbol.

Figure 5 shows representative results from select inhibition titrations from eight assays used in the study. In some cases (RSV and human metapneumovirus), using lower concentrations of solids led to non-detects due to loss of assay sensitivity. For 5 of the assays (RSV, HMPV, SARS-CoV-2 N and S, influenza A), lower concentrations of solids resulted in non-detects, or the same or slightly lower concentrations than those measured with 75 mg/ml. For the remaining three assays, the concentrations obtained using solids concentrations lower than 75 mg/ml were inconsistent - some were higher and some were lower. In all cases, the differences in concentrations was at most about 2X. Overall, we interpret these results to indicate that inhibition is not significantly affecting our analyses and the choice of using 75 mg/ml effectively balances alleviating inhibition while providing good sensitivity.

## Usage Notes

Environmental data exhibit variability that is caused by different factors than those that cause variability in clinical case or syndromic data. Viral nucleic acid concentrations in wastewater may vary owing to a number of reasons. First, the sources of nucleic acids (from human shedding) are not constant with time. The amount of viral nucleic acids deposited in the sewage system may change based on movement of people into and out of the sewershed, intermittent deliveries of septic wastes to the system, or the time course of shedding by infected individuals, among other factors. Second, wastewater solids from which these data are derived may have variable concentrations spatially within the material that is sampled - there are “clumps” that may contain more or less viral nucleic acids. Third, analytic measurement variability is also present. Fourth, concentrations of viral nucleic acids that individuals shed differ from person to person and introduce stochastic variability into the results. Despite this variability, wastewater data have been shown to be highly correlated with measures of disease occurrence in sewersheds^5,12–14,22^. To address variability in this study, we processed high frequency samples collected daily, and ran 10 replicates and multiple controls to describe analytical error and ensure high quality measurements. When we share these data with stakeholders, we typically normalize viral nucleic acid concentrations by concentrations of PMMoV RNA to account for process variability and the fecal strength of the wastewater^10,11^, and use 5-d trimmed average smoothing of the high-frequency data to reduce the influence of measurement outliers.

Some of the data described in this data descriptor has been analyzed in previous publications including Wolfe *et al*.^22^, Wolfe *et al*.^30^, Yu *et al*.^31^, Boehm *et al*.^32^, and Wolfe *et al*.^12^. However, most of the data in this descriptor has not been analyzed.

## Supplementary information

Supplementary Information

## Data Availability

The plots made in this Data Descriptor were generated using IGOR PRO v8 (Wavemetrics, Lake Oswego, Oregon).
