# Supplementary material for: Human viral nucleic acids concentrations in wastewater solids from Central and Coastal California USA
Source: Sci Data. 2023 Jun 22;10:396. doi: 10.1038/s41597-023-02297-7 (PMC10287720; doi:10.1038/s41597-023-02297-7)
Supplement: Supplementary file 1 — Supplementary Information [file 41597_2023_2297_MOESM1_ESM.pdf]

Supplementary Information

Human viral nucleic acids concentrations in wastewater solids from Central and Coastal California, USA

Authors

Alexandria B. Boehm[1], Marlene K. Wolfe[2], Krista R. Wigginton[3], Amanda Bidwell[1], Bradley White[4], Bridgette Hughes[4], Dorothea Duong[4], Vikram Chan-Herur[4], Heather N. Bischel[5], Colleen C. Naughton[6]

Affiliations

1. Department of Civil & Environmental Engineering, School of Engineering and Doerr School of Sustainability, Stanford University, Stanford, CA, USA
2. Gangarosa Department of Environmental Health, Rollins School of Public Health, Emory University, Atlanta, GA, USA
3. Department of Civil and Environmental Engineering, University of Michigan, Ann Arbor 48109, Michigan, USA
4. Verily Life Sciences, South San Francisco, CA, USA
5. Department of Civil and Environmental Engineering, University of California Davis, Davis, CA 95616, United States
6. Department of Civil and Environmental Engineering, University of California Merced, Merced, CA 95343, United States

corresponding author: Alexandria Boehm (aboehm@stanford.edu)

Table of contents

Figure S1 on page 2

Table S1 on page 3

Table S2 on page 6

References on page 9

Environmental Microbiology Minimum Information Checklist

Study Description

Study: SCAN
Date: March 2023
Completed by: Alexandria Boehm

Environmental Sampling	Sample Treatment	Sample Reduction	Nucleic Acid Extraction	Reverse Transcription	PCR Detection	Analysis
Decribed in methods section	☐ Performed<br>No sample treatment performed	■ Performed<br>Centrifugation was used, as described in the methods	Methods provided in the Data Descriptor.	■ Performed<br>One Step RT-PCR	☐ qPCR ■ dPCR<br>All methods provided including the dMIE checklist	No formal analysis was done in this project

Control Checklist

	Environmental Sampling	Sample Treatment	Sample Reduction	Nucleic Acid Extraction	Reverse Transcription	PCR Detection
Step performed	■	☐	■	☐	■	■
**Negative Controls**						
Step has control info	☐	☐	☐	■	■	■
# control replicates	0	0	0	3-7	3-7	3-7
Control result reported	☐	☐	☐	■	■	■
Data handling reported	■	☐	■	■	■	■
**Positive Controls**						
Control introduced	☐	☐	■	☐	☐	☐
Internal/External	N/A	N/A	Internal	External	External	External
Independent/Parallel	N/A	N/A	Parallel	Independent	Independent	Independent
Step has control info	☐	☐	■	■	■	■
# control replicates	0	0	10	1	1	1
Control result reported	☐	☐	■	■	■	■
Data Handling reported	☐	☐	■	■	■	■

Process Checklist

Environmental Sampling
- ■ Sampling Procedure
- ☐ Number of samples
- ■ Sample amount, mean, range
- ■ Sampling locations, dates, times

Sample Treatment
- ☐ Performed
- ☐ Treatment procedure
- ☐ Reagents

Sample Reduction
- ☐ Performed
- ■ Reduction procedure
- ☐ Reagents
- ☐ Concentration Factor

Nucleic Acid Extraction
- ■ Extraction procedure
- ■ Amount extracted, amount obtained
- ■ Extract storage conditions

qPCR or dPCR
- ■ Target gene name, amplicon length
- ■ Thermocycling temperatures and times
- ■ Master mix: composition, vendors, concentrations
- ■ Additives: vendors, concentrations
- ■ Template amount added, pre-treatment (if any)
- ■ Primers: sequences, concentrations, vendors, references
- ■ Amplicon confirmation method (probe, melt curve, etc)
- ■ Probe sequence, concentration, vendor, reference
- ■ Instrumentation
- ☐ Equivalent volume of sample analyzed by PCR
- ■ Inhibition assessment procedure
- ☐ Inhibition control description (if used)
- ■ Number samples tested and found inhibited

Reverse Transcription
- ■ Performed
- ■ One or two step
- ☐ cDNA storage conditions (if two step)
- ■ Reaction temperatures and times
- ■ Reaction reagents and concentrations
- ■ Priming method
- ■ Reaction volume, added template amount
- ■ Inhibition assessment procedure
- ☐ Inhibition control description (if used)
- ■ Number samples tested and found inhibited

Analysis – dPCR
- ■ Threshold settings
- ■ Technical replicates, number, well merging
- ■ Partitions measured, number, mean, variance
- ■ Partition volume
- ■ Target copies per partition, mean, variance
- ■ Program used for dPCR analysis
- ■ Explanation of control results, example plots

Analysis – qPCR
- ☐ Method for handling failed negative controls
- ☐ Technical replicates, number, calculations
- ☐ Calibration standards: description and source
- ☐ Method of quantifying standards
- ☐ Calibration curve slope
- ☐ Calibration curve R2
- ☐ Lowest standard measured or 95% LOD
- ☐ Cq value determination method

Figure S1. EMMI checklist[1]

Table S1. Forward and reverse primers, and probe sequences for detection of viral nucleic acids in this study. Primers and probes were purchased from Integrated DNA Technologies (IDT, Coralville, Iowa). All probes contained fluorescent molecules and quenchers (5' FAM and/or HEX/ZEN/3' IBFQ). FAM, 6-fluorescein amidite; HEX, hexachloro-fluorescein; ZEN, a proprietary internal quencher from IDT; IBFQ, Iowa Black FQ. The references for the primers and probes are provided in Table 2. The SARS-CoV-2 S gene assay was changed twice during the study to accommodate mutations in new circulating variants.

Target	Primer/Probe	Sequence
SARS-CoV-2 N Gene	Forward	CATTACGTTTGGTGGACCCT
	Reverse	CCTTGCCATGTTGAGTGAGA
	Probe	CGCGATCAAAACAACGTCGG
SARS CoV-2 S Gene (used through 9/4/21)	Forward	CAGACTAATTCTCCTCGGCG
	Reverse	TGCACCAAGTGACATAGTGT
	Probe	AGCTAGTCAATCCATCATTGCCT
SARS CoV-2 S gene (rev) (used 9/4/21-12/22/21)	Forward	CAGACTAATTCTCSTCGGCG
	Reverse	TGCACCAAGTGACATAGTGT
	Probe	AGCTAGTCAATCCATCATTGCCT
SARS CoV-2 S gene (rev2) (used 12/22/21-12/31/22)	Forward	CAGACTAAKTCTCVTCGGCG
	Reverse	TGCACCAAGTGACATAGTGT
	Probe	AGCTAGTCAATCCATCATTGCCT
SARS-CoV-2 ORF1a	Forward	CAGAACTGGAACCACCTTGT
	Reverse	TACAGTTGAATTGGCAGGCA
	Probe	TGCCACAGTACGTCTACAAGC
BCoV	Forward	CTGGAAGTTGGTGGAGTT
	Reverse	ATTATCGGCCTAACATACATC
	Probe	CCTTCATATCTATACACATCAAGTTGTT
PMMoV	Forward	GAGTGGTTTGACCTTAACGTTTGA
	Reverse	TTGTCGGTTGCAATGCAAGT

	Probe	CCTACCGAAGCAAATG
SARS-CoV-2 HV69-70	Forward	ACTCAGGACTTGTTCTTACCT
	Reverse	TGGTAGGACAGGGTTATCAAAC
	Probe	ATGCTATCTCTGGGACCAAT
SARS-CoV-2 del156-157	Forward	ATTCGAAGACCCAGTCCCTA
	Reverse	AGGTCCATAAGAAAAGGCTGA
	Probe	TGGATGGAAAGTGGAGTTTATTCTAG
SARS-CoV-2 Del143-145 Omicron BA.1 mutation	Forward	ATTCGAAGACCCAGTCCCTA
	Reverse	ACTCTGAACTCACTTTCCATCC
	Probe	TTGTAATGATCCATTTTTGGACCACAA
SARS-CoV-2 LPPA24S Mutations in BA.2, BA.4. and BA.5	Forward	GCCACTAGTCTCTAGTCAGTGTG
	Reverse	TGTCAGGGTAATAAACACCACGT
	Probe	CAGAACTCAATCATACACTAATTCTTTCAC
SARS-CoV-2 BA.4 mutations	Forward	TAATAAAGGAGCTGGTGGCC
	Reverse	ATGAGTTCACGGGTAACACC
	Probe	CGGCGCCGATCTAGACTTAG
SARS-CoV-2 BA.2.75 Mutations in BA.2.75	Forward	CGAAGACCCAGTCCCTACTT
	Reverse	TCCATAAGAAAAGGCTGAGAGA
	Probe	CCACGAAAACAACAAAAGTCGGATG
Influenza A	Forward	CAAGACCAATCYTGTCACCTCTGAC
	Reverse	GCATTYTGGACAAAVCGTCTACG
	Probe	TGCAGTCCTCGCTCACTGGGCACG

Influenza B	Forward	TCCTCAAYTCACTCTTCGAGCG
	Reverse	CGGTGCTCTTGACCAAATTGG
	Probe	CCAATTCGAGCAGCTGAAACTGCGGTG
RSV	Forward	CTCCAGAATAYAGGCATGAYTCTCC
	Reverse	GCYCTYCTAATYACWGCTGTAAGAC
	Probe	TAACCAAATTAGCAGCAGGAGATAGATCAG
HMPV	Forward	ACTTTATTGGAGAAGGAGCAGG
	Reverse	GGGTAATGRTGATCAAGRTCA
	Probe	AYTGGATGGCMAGAACAGCA
Norovirus GII	Forward	ATGTTCAGRTGGATGAGRTTCTCWGA
	Reverse	TCGACGCCATCTTCATTCACA
	Probe	AGCACGTGGGAGGGCGATCG
MPOX G2R_G (used through 11/30/22)	Forward	GGAAAATGTAAAGACAACGAATACAG
	Reverse	GCTATCACATAATCTGGAAGCGTA
	Probe	AAGCCGTAATCTATGTTGTCTATCGTGTCC
MPOX G2R_WA (used 12/1/22-12/31/22)	Forward	CACACCGTCTCTTCCACAGA
	Reverse	GATACAGGTTAATTTCCACATCG
	Probe	AACCCGTCGTAACCAGCAATACATTT

ITEM TO CHECK	PROVIDED	COMMENT
	**Y/N**	
**1. SPECIMEN**		
Detailed description of specimen type and numbers	**Y**	Methods
Sampling procedure (including time to storage)	**Y**	Methods
Sample aliquotation, storage conditions and duration	**Y**	Methods
**2. NUCLEIC ACID EXTRACTION**		
Description of extraction method including amount of sample processed	**Y**	Methods
Volume of solvent used to elute/resuspend extract	**Y**	Methodst
Number of extraction replicates	**Y**	Methods
Extraction blanks included?	**Y**	Methods
**3. NUCLEIC ACID ASSESSMENT AND STORAGE**		
Method to evaluate quality of nucleic acids	**N**	Not Done
Method to evaluate quantity of nucleic acids (including molecular weight and calculations when using mass)	**N**	Not Done
Storage conditions: temperature, concentration, duration, buffer, aliquots	**Y**	Methods
Clear description of dilution steps used to prepare working DNA solution	**Y**	Methods
**4. NUCLEIC ACID MODIFICATION**		
Template modification (digestion, sonication, pre-amplification, bisulphite etc.)	**N**	NA

Details of repurification following modification if performed	**Y**	Zymo Column in Methods
**5. REVERSE TRANSCRIPTION**		
cDNA priming method and concentration	**N**	NA
One or two step protocol (include reaction details for two step)	**Y**	Methods
Amount of RNA added per reaction	**Y**	Methods t
Detailed reaction components and conditions	**Y**	Methods
Estimated copies measured with and without addition of RT*	**N**	Not Done
Manufacturer of reagents used with catalogue and lot numbers	**Y**	Lot Numbers are Not Reported
Storage of cDNA: temperature, concentration, duration, buffer and aliquots	**N**	NA
**6. dPCR OLIGONUCLEOTIDES DESIGN AND TARGET INFORMATION**		
Sequence accession number or official gene symbol	**N**	Located in references for each assay
Method (software) used for design and *in silico* verification	**N**	Located in references for each assay
Location of amplicon	**S**	Also located in references for each assay
Amplicon length	**N**	Located in references for each assay
Primer and probe sequences (or amplicon context sequence)**	**Y**	Methods
Location and identity of any modifications	**N**	NA
Manufacturer of oligonucleotides	**Y**	Methods
**7. dPCR PROTOCOL**		
Manufacturer of dPCR instrument and instrument model	**Y**	Methods

Buffer/kit manufacturer with catalogue and lot number	**Y**	Methods
Primer and probe concentration	**Y**	Methods
Pre-reaction volume and composition (incl. amount of template and if restriction enzyme added)	**Y**	Methods
Template treatment (initial heating or chemical denaturation)	**N**	NA
Polymerase identity and concentration, Mg++ and dNTP concentrations***	**N**	Included in Kit Manuals
Complete thermocycling parameters	**Y**	Methods
**8. ASSAY VALIDATION**		
Details of optimisation performed	**N**	Commercial Kit, Followed Manufacturer's Instructions
Analytical specificity (vs. related sequences) and limit of blank (LOB)	**N**	We chose to report LOD (below)
Analytical sensitivity/LoD and how this was evaluated	**Y**	Methods
Testing for inhibitors (from biological matrix/extraction)	**Y**	Methods
**9. DATA ANALYSIS**		
Description of dPCR experimental design	**Y**	Methods
Comprehensive details negative and positive of controls (whether applied for QC or for estimation of error)	**Y**	Methods
Partition classification method (thresholding)	**Y**	Methods
Examples of positive and negative experimental results (including fluorescence plots in supplemental material)	**Y**	Provided in references
Description of technical replication	**Y**	Methods
Repeatability (intra-experiment variation)	**Y**	Methods

Reproducibility (inter-experiment/user/lab etc. variation)	**N**	Assays were only completed within one laboratory
Number of partitions measured (average and standard deviation)	**Y**	Methods
Partition volume	**Y**	Reported by Manufacturer
Copies per partition (λ or equivalent) (average and standard deviation)	**Y**	Methods
dPCR analysis program (source, version)	**Y**	Methods t
Description of normalisation method	**N**	NA
Statistical methods used for analysis	**N**	NA
Data transparency	raw data uploaded to online repository with ID:	Website to access data provided in main text, data uploaded to stanford digital repository

Table S2. dMIQE2020 checklist for authors, reviewers and editors from Huggett et al.[2].

1. Borchardt, M. A. *et al.* The Environmental Microbiology Minimum Information (EMMI) Guidelines: qPCR and dPCR Quality and Reporting for Environmental Microbiology. *Environ. Sci. Technol.* **55**, 10210–10223 (2021).
2. The dMIQE Group & Huggett, J. F. The Digital MIQE Guidelines Update: Minimum Information for Publication of Quantitative Digital PCR Experiments for 2020. *Clinical Chemistry* **66**, 1012–1029 (2020).
